# (1*E*,4*E*)-1,5-Bis(2,4,5-trimethoxy­phen­yl)penta-1,4-dien-3-one^1^

**DOI:** 10.1107/S1600536809055421

**Published:** 2010-01-09

**Authors:** Hoong-Kun Fun, Pumsak Ruanwas, Suchada Chantrapromma

**Affiliations:** a X-ray Crystallography Unit, School of Physics, Universiti Sains Malaysia, 11800 USM, Penang, Malaysia; b Crystal Materials Research Unit, Department of Chemistry, Faculty of Science, Prince of Songkla University, Hat-Yai, Songkhla 90112, Thailand

## Abstract

There are three mol­ecules in the asymmetric unit of the title compound, C_23_H_26_O_7_, in which the dihedral angles between two benzene rings are 4.34 (9), 18.11 (8) and 8.54 (8)°. The central penta-1,4-dien-3-one fragment makes dihedral angles of 3.95 (9) and 3.32 (16)° with the two adjacent benzene rings in one mol­ecule, whereas the corresponding pairs of angles in the other two mol­ecules are 10.34 (9)/17.46 (8)° and 7.87 (8)/13.33 (8)°. In the crystal, mol­ecules are linked by inter­molecular C—H⋯O and C—H⋯π weak inter­actions into a three-dimensional network. Finally, π–π inter­actions [centroid⋯centroid distances = 3.5984 (10) and 3.5545 (10) Å] are observed.

## Related literature

For bond-length data, see: Allen *et al.* (1987 ▶). For hydrogen-bond motifs, see: Bernstein *et al.* (1995 ▶). For a related structure, see: Harrison *et al.* (2006 ▶). For background to and applications of chalcones, see: Baeyer & von Villiger (1902 ▶); Gomes *et al.* (2009 ▶); Gould *et al.* (1995 ▶); Masuda *et al.* (1993 ▶); Quincoces *et al.* (2002 ▶; 2003 ▶; 2008 ▶); Uchida *et al.* (1998 ▶). For the stability of the temperature controller, see: Cosier & Glazer, (1986 ▶).
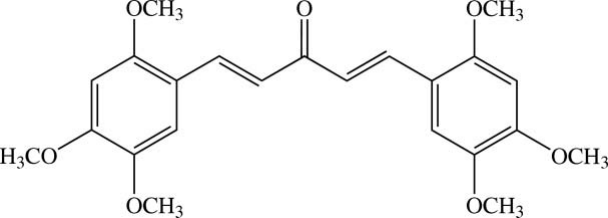


## Experimental

### 

#### Crystal data

C_23_H_26_O_7_
*M* *_r_* = 414.44
Monoclinic, 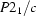

*a* = 9.4157 (1) Å
*b* = 36.8613 (5) Å
*c* = 19.1226 (3) Å
β = 107.737 (1)°
*V* = 6321.49 (15) Å^3^
*Z* = 12
Mo *K*α radiation
μ = 0.10 mm^−1^
*T* = 100 K
0.37 × 0.22 × 0.13 mm

#### Data collection

Bruker APEXII CCD area-detector diffractometer
Absorption correction: multi-scan (*SADABS*; Bruker, 2005 ▶) *T*_min_ = 0.965, *T*_max_ = 0.988
81495 measured reflections
18387 independent reflections
12498 reflections with *I* > 2σ(*I*)
*R*_int_ = 0.046

#### Refinement

*R*[*F* ^2^ > 2σ(*F* ^2^)] = 0.067
*wR*(*F* ^2^) = 0.150
*S* = 1.06
18387 reflections
829 parameters
H-atom parameters constrained
Δρ_max_ = 0.38 e Å^−3^
Δρ_min_ = −0.26 e Å^−3^

### 

Data collection: *APEX2* (Bruker, 2005 ▶); cell refinement: *SAINT* (Bruker, 2005 ▶); data reduction: *SAINT*; program(s) used to solve structure: *SHELXTL* (Sheldrick, 2008 ▶); program(s) used to refine structure: *SHELXTL*; molecular graphics: *SHELXTL*; software used to prepare material for publication: *SHELXTL* and *PLATON* (Spek, 2009 ▶).

## Supplementary Material

Crystal structure: contains datablocks global, I. DOI: 10.1107/S1600536809055421/hb5282sup1.cif

Structure factors: contains datablocks I. DOI: 10.1107/S1600536809055421/hb5282Isup2.hkl

Additional supplementary materials:  crystallographic information; 3D view; checkCIF report

## Figures and Tables

**Table 1:** Hydrogen-bond geometry (Å, °) *Cg*3 and *Cg*4 are the centroids of the C1*C*–C6*C* and C12*C*–C17*C* rings, respectively.

*D*—H⋯*A*	*D*—H	H⋯*A*	*D*⋯*A*	*D*—H⋯*A*
C10*B*—H10*B*⋯O1*C*^i^	0.93	2.29	3.149 (2)	153
C10*C*—H10*C*⋯O1*B*	0.93	2.33	3.195 (2)	155
C14*B*—H14*B*⋯O1*A*^ii^	0.93	2.52	3.353 (2)	149
C21*A*—H21*B*⋯O3*A*^iii^	0.96	2.49	3.301 (2)	142
C22*A*—H22*C*⋯O7*C*^iv^	0.96	2.50	3.435 (2)	165
C22*B*—H22*D*⋯O2*A*^v^	0.96	2.50	3.407 (2)	158
C22*B*—H22*F*⋯O1*A*^ii^	0.96	2.58	3.227 (2)	125
C23*A*—H23*A*⋯O5*A*^i^	0.96	2.52	3.308 (2)	140
C23*A*—H23*C*⋯O3*C*	0.96	2.53	3.452 (2)	161
C23*C*—H23*G*⋯O5*C*^i^	0.96	2.54	3.305 (2)	136
C18*C*—H18*H*⋯*Cg*4^ii^	0.96	2.80	3.678 (2)	152
C20*A*—H20*B*⋯*Cg*3^vi^	0.96	2.94	3.855 (2)	159

Symmetry codes: (i) 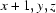; (ii) 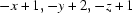; (iii) 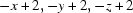; (iv) 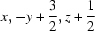; (v) 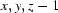; (vi) 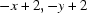, 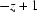.

**Fig. 1.**
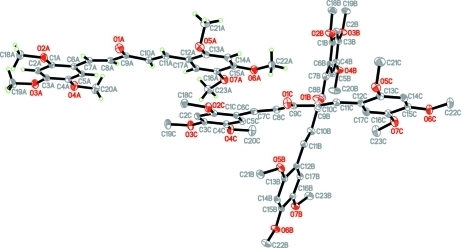
The molecular structure of (I) showing 50% probability displacement ellipsoids. H atoms of molecules B and C are omitted for clarity.

**Fig. 2.**
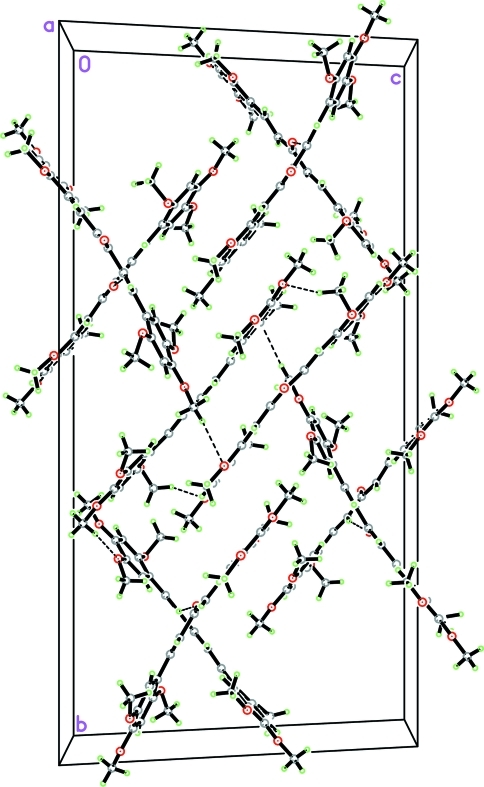
The crystal packing of (I) viewed along the a axis, showing the three-dimensional network. Hydrogen bonds are shown as dashed lines.

## supplementary crystallographic information

### Comment

Bischalcones with the general formula Ar—CH=CH—CO—CH=CH—Ar (Baeyer & Villiger, 1902) are an important class of compounds because they are widely used in many fields such as in organic solid-state photochemistry (Gould *et al.*, 1995), anti-oxidative and anti-inflammatory activities (Masuda *et al.*, 1993), cytotoxicity (Quincoces *et al.*, 2002; 2003; 2008) and activities involving their non-linear optical (Uchida *et al.*, 1998) and fluorescence properties (Gomes *et al.*, 2009). However not much crystal structures of this type of compound were reported. The title bischalcone (I) was synthesized on the account of its fluorescence property which will be reported elsewhere together with the other bichalcone derivatives. We reported herein the crystal structure of (I).

There are three crystallographic independent molecules *A*, *B* and *C* in the asymmetric unit of (I) (Fig. 1) with slight differences in bond angles. The molecular structure of (I), C_23_H_26_O_7_, is not planar. The dihedral angle between the C1–C6 and C12–C17 benzene rings is 4.34 (9)° in molecule *A* whereas its is 18.11 (8) and 8.54 (8)° in molecules *B* and *C*, respectively. The central penta-1,4-dien-3-one fragment (C7–C11/O1) is planar with the r.m.s. 0.0204 (2) Å for molecule *A* [0.0227 (2) and 0.0252 (2) Å for molecule *B* and *C*, respectively]. This fragment makes the dihedral angles of 3.95 (9) and 3.32 (16)° with the two adjacent C1–C6 and C12–C17 benzene rings, respectively in molecule *A* whereas the corresponding values are 10.34 (9) and 17.46 (8)° in molecule *B*; and 7.87 (8) and 13.33 (8)° in the molecule *C*. The three methoxy groups on C1–C6 benzene ring are essentially planarly attached [C18–O2–C1–C2, C19–O3–C3–C2 and C20–O4–C4–C5 torsion angles of -3.7 (2), 1.4 (3) and -3.9 (2)° in molecule *A*; -0.2 (3), 3.5 (3) and -1.7 (2)° in molecule *B*; 3.8 (3), 4.4 (3) and -4.3 (3)° in molecule *C*]. The middle methoxy group of the 2,4,5-trimethoxyphenyl moeity is co-planar with the C12–C17 benzene ring, with the C22–O6–C15–C14 torsion angle being 1.1 (3)° whereas the other two methoxy groups are twisted with the torsion angles C21–O5–C13–C14 and C23–O7–C16–C17 being -111.48 (18) and -14.4 (3)°, respectively in molecule *A* [the three corresponding values are -7.6 (3), -30.2 (2) and 9.6 (2)° in molecule *B* and 0.4 (3), 73.5 (2) and -8.2 (3)° in molecule *C*]. Intramolecular C—H···O weak interactions (Table 1) generate S(5) ring motifs (Bernstein *et al.*, 1995). The bond distances agree with the literature values (Allen *et al.*, 1987) and are comparable with the related structure (Harrison *et al.*, 2006).

In the crystal packing (Fig. 2), the molecules are linked by intermolecular C—H···O weak interactions (Table 1) into a supramolecular three-dimensional network. The crystal is stabilized by intra- and intermolecular C—H···O weak interactions and C—H···π interactions (Table 1). π–π interactions were observed with the distances of *Cg*_1_···*Cg*_4_ = 3.5984 (10) Å (symmetry code: *x*, *y*, *z*) and *Cg*_2_···*Cg*_5_ = 3.5984 (10) Å (symmetry code: 1 + *x*, 3/2 - *y*, 1/2 + *z*); *Cg*_1_, *Cg*_2_, *Cg*_3_, *Cg*_4_ and *Cg*_5_ are the centroids of C12A–C17A, C1B–C6B, C12B–C17B, C1C–C6C and C12C–C17B rings, respectively.

### Experimental

The title compound was synthesized by dissolving 2,4,5-trimethoxybenzaldehyde (0.5 g, 4.85 mmol) in acetone (50 ml). NaOH 50% aqueous solution (2 ml) was then added. After stirring at room temperature for 1 hr, the resulting orange solid was collected by filtration, washed with distilled water and dried. Pale yellow blocks of (I) were recrystalized from acetone/ethanol (1:1 *v*/*v*) by the slow evaporation of the solvent at room temperature after a week, Mp. 441–442 K.

### Refinement

All H atoms were positioned geometrically and allowed to ride on their parent atoms, with C—H = 0.93 Å for aromatic and CH; 0.96 Å for CH_3_ atoms. The *U*_iso_ values were constrained to be 1.5*U*_eq_ of the carrier atom for methyl H atoms and 1.2*U*_eq_ for the remaining H atoms. A rotating group model was used for the methyl groups. The highest residual electron density peak is 0.71 Å from C12C and the deepest hole is 0.45 Å from C9A.

### Crystal data

**Table:**

C_23_H_26_O_7_	*F*(000) = 2640
*M**_r_* = 414.44	*D*_x_ = 1.306 Mg m^−^^3^
Monoclinic, *P*2_1_/*c*	Melting point = 441–442 K
Hall symbol: -P 2ybc	Mo *K*α radiation, λ = 0.71073 Å
*a* = 9.4157 (1) Å	Cell parameters from 18387 reflections
*b* = 36.8613 (5) Å	θ = 1.1–30.0°
*c* = 19.1226 (3) Å	µ = 0.10 mm^−^^1^
β = 107.737 (1)°	*T* = 100 K
*V* = 6321.49 (15) Å^3^	Block, colorless
*Z* = 12	0.37 × 0.22 × 0.13 mm

### Data collection

**Table:**

Bruker APEXII CCD area-detector diffractometer	18387 independent reflections
Radiation source: sealed tube	12498 reflections with *I* > 2σ(*I*)
graphite	*R*_int_ = 0.046
phi and ω scans	θ_max_ = 30.0°, θ_min_ = 1.1°
Absorption correction: multi-scan (*SADABS*; Bruker, 2005)	*h* = −13→13
*T*_min_ = 0.965, *T*_max_ = 0.988	*k* = −45→51
81495 measured reflections	*l* = −26→26

### Refinement

**Table:**

Refinement on *F*^2^	Primary atom site location: structure-invariant direct methods
Least-squares matrix: full	Secondary atom site location: difference Fourier map
*R*[*F*^2^ > 2σ(*F*^2^)] = 0.067	Hydrogen site location: inferred from neighbouring sites
*wR*(*F*^2^) = 0.150	H-atom parameters constrained
*S* = 1.06	*w* = 1/[σ^2^(*F*_o_^2^) + (0.0517*P*)^2^ + 4.2947*P*] where *P* = (*F*_o_^2^ + 2*F*_c_^2^)/3
18387 reflections	(Δ/σ)_max_ = 0.002
829 parameters	Δρ_max_ = 0.38 e Å^−^^3^
0 restraints	Δρ_min_ = −0.25 e Å^−^^3^

### Special details

**Table:**

Experimental. The crystal was placed in the cold stream of an Oxford Cryosystems Cobra open-flow nitrogen cryostat (Cosier & Glazer, 1986) operating at 100.0 (1) K.
Geometry. All e.s.d.'s (except the e.s.d. in the dihedral angle between two l.s. planes) are estimated using the full covariance matrix. The cell e.s.d.'s are taken into account individually in the estimation of e.s.d.'s in distances, angles and torsion angles; correlations between e.s.d.'s in cell parameters are only used when they are defined by crystal symmetry. An approximate (isotropic) treatment of cell e.s.d.'s is used for estimating e.s.d.'s involving l.s. planes.
Refinement. Refinement of *F*^2^ against ALL reflections. The weighted *R*-factor *wR* and goodness of fit *S* are based on *F*^2^, conventional *R*-factors *R* are based on *F*, with *F* set to zero for negative *F*^2^. The threshold expression of *F*^2^ > σ(*F*^2^) is used only for calculating *R*-factors(gt) *etc*. and is not relevant to the choice of reflections for refinement. *R*-factors based on *F*^2^ are statistically about twice as large as those based on *F*, and *R*- factors based on ALL data will be even larger.

### Fractional atomic coordinates and isotropic or equivalent isotropic displacement parameters (Å^2^)

**Table:**

	*x*	*y*	*z*	*U*_iso_*/*U*_eq_	
O1A	0.61853 (14)	0.99343 (4)	0.88798 (8)	0.0259 (3)	
O2A	0.77349 (15)	1.09843 (4)	1.05130 (7)	0.0262 (3)	
O3A	1.27431 (15)	1.14769 (4)	1.10886 (7)	0.0279 (3)	
O4A	1.34759 (15)	1.09825 (4)	1.03136 (7)	0.0274 (3)	
O5A	0.47925 (14)	0.89473 (3)	0.70474 (7)	0.0242 (3)	
O6A	0.83737 (14)	0.84534 (3)	0.59545 (7)	0.0238 (3)	
O7A	1.03708 (14)	0.89136 (4)	0.66503 (8)	0.0257 (3)	
C1A	0.9167 (2)	1.09853 (5)	1.04751 (10)	0.0203 (4)	
C2A	1.0225 (2)	1.12410 (5)	1.08416 (10)	0.0225 (4)	
H2AA	0.9977	1.1416	1.1134	0.027*	
C3A	1.1646 (2)	1.12340 (5)	1.07698 (10)	0.0218 (4)	
C4A	1.2038 (2)	1.09658 (5)	1.03338 (10)	0.0212 (4)	
C5A	1.0980 (2)	1.07165 (5)	0.99736 (10)	0.0198 (4)	
H5AA	1.1233	1.0542	0.9681	0.024*	
C6A	0.95201 (19)	1.07169 (5)	1.00345 (9)	0.0189 (3)	
C7A	0.84093 (19)	1.04502 (5)	0.96501 (9)	0.0191 (3)	
H7AA	0.7457	1.0472	0.9695	0.023*	
C8A	0.86455 (19)	1.01757 (5)	0.92372 (9)	0.0184 (3)	
H8AA	0.9607	1.0138	0.9214	0.022*	
C9A	0.74500 (19)	0.99314 (5)	0.88200 (9)	0.0182 (3)	
C10A	0.78751 (19)	0.96863 (5)	0.83046 (10)	0.0193 (3)	
H10A	0.8828	0.9706	0.8260	0.023*	
C11A	0.69497 (19)	0.94367 (5)	0.78996 (10)	0.0190 (3)	
H11A	0.5998	0.9424	0.7949	0.023*	
C12A	0.72921 (19)	0.91817 (5)	0.73880 (9)	0.0180 (3)	
C13A	0.62264 (18)	0.89351 (5)	0.69924 (10)	0.0192 (3)	
C14A	0.65461 (19)	0.86840 (5)	0.65119 (10)	0.0201 (4)	
H14A	0.5822	0.8519	0.6260	0.024*	
C15A	0.79434 (19)	0.86815 (5)	0.64133 (10)	0.0191 (3)	
C16A	0.90353 (19)	0.89331 (5)	0.67985 (10)	0.0191 (3)	
C17A	0.87092 (19)	0.91748 (5)	0.72729 (10)	0.0191 (3)	
H17A	0.9437	0.9338	0.7526	0.023*	
C18A	0.7343 (2)	1.12439 (6)	1.09820 (11)	0.0297 (4)	
H18A	0.6322	1.1209	1.0964	0.045*	
H18B	0.7469	1.1485	1.0819	0.045*	
H18C	0.7976	1.1212	1.1477	0.045*	
C19A	1.2386 (2)	1.17568 (5)	1.15221 (11)	0.0315 (5)	
H19A	1.3210	1.1922	1.1683	0.047*	
H19B	1.2186	1.1651	1.1942	0.047*	
H19C	1.1520	1.1886	1.1233	0.047*	
C20A	1.3885 (2)	1.07307 (6)	0.98420 (12)	0.0319 (5)	
H20A	1.4908	1.0769	0.9867	0.048*	
H20B	1.3260	1.0766	0.9346	0.048*	
H20C	1.3762	1.0488	0.9995	0.048*	
C21A	0.4405 (2)	0.86423 (5)	0.74166 (11)	0.0258 (4)	
H21A	0.3344	0.8636	0.7323	0.039*	
H21B	0.4888	0.8664	0.7935	0.039*	
H21C	0.4723	0.8423	0.7239	0.039*	
C22A	0.7279 (2)	0.82026 (5)	0.55365 (11)	0.0271 (4)	
H22A	0.7709	0.8055	0.5240	0.041*	
H22B	0.6445	0.8334	0.5225	0.041*	
H22C	0.6949	0.8051	0.5864	0.041*	
C23A	1.1351 (2)	0.92146 (5)	0.68810 (12)	0.0271 (4)	
H23A	1.2172	0.9192	0.6685	0.041*	
H23B	1.1720	0.9220	0.7407	0.041*	
H23C	1.0821	0.9435	0.6706	0.041*	
O1B	0.67886 (14)	0.79921 (4)	0.36869 (8)	0.0296 (3)	
O2B	0.90239 (14)	0.70489 (4)	0.53331 (8)	0.0273 (3)	
O3B	1.41632 (14)	0.66614 (4)	0.58013 (7)	0.0267 (3)	
O4B	1.45884 (14)	0.71537 (4)	0.49398 (8)	0.0297 (3)	
O5B	0.52194 (14)	0.92198 (3)	0.26449 (7)	0.0223 (3)	
O6B	0.84488 (14)	0.99180 (3)	0.15955 (7)	0.0236 (3)	
O7B	1.04120 (13)	0.94003 (3)	0.19064 (7)	0.0213 (3)	
C1B	1.04099 (19)	0.70706 (5)	0.52494 (10)	0.0201 (4)	
C2B	1.1587 (2)	0.68375 (5)	0.56056 (10)	0.0202 (4)	
H2BA	1.1447	0.6659	0.5921	0.024*	
C3B	1.2953 (2)	0.68751 (5)	0.54854 (10)	0.0209 (4)	
C4B	1.3190 (2)	0.71448 (5)	0.50116 (10)	0.0220 (4)	
C5B	1.20241 (19)	0.73714 (5)	0.46616 (10)	0.0204 (4)	
H5BA	1.2170	0.7549	0.4346	0.024*	
C6B	1.06154 (19)	0.73390 (5)	0.47726 (10)	0.0191 (3)	
C7B	0.93671 (19)	0.75743 (5)	0.44029 (10)	0.0195 (3)	
H7BA	0.8464	0.7524	0.4486	0.023*	
C8B	0.93721 (19)	0.78538 (5)	0.39580 (10)	0.0201 (4)	
H8BA	1.0248	0.7913	0.3856	0.024*	
C9B	0.80148 (18)	0.80706 (5)	0.36257 (10)	0.0183 (3)	
C10B	0.82631 (19)	0.83942 (5)	0.32294 (10)	0.0199 (4)	
H10B	0.9200	0.8424	0.3169	0.024*	
C11B	0.72314 (18)	0.86483 (5)	0.29492 (9)	0.0170 (3)	
H11B	0.6277	0.8612	0.2983	0.020*	
C12B	0.75129 (18)	0.89799 (4)	0.25939 (9)	0.0154 (3)	
C13B	0.64957 (18)	0.92681 (5)	0.24499 (9)	0.0164 (3)	
C14B	0.67679 (19)	0.95861 (5)	0.21126 (10)	0.0183 (3)	
H14B	0.6070	0.9773	0.2009	0.022*	
C15B	0.80799 (19)	0.96226 (5)	0.19337 (9)	0.0170 (3)	
C16B	0.91373 (18)	0.93376 (5)	0.20913 (9)	0.0169 (3)	
C17B	0.88319 (18)	0.90239 (5)	0.24028 (9)	0.0171 (3)	
H17B	0.9517	0.8835	0.2490	0.021*	
C18B	0.8747 (2)	0.67823 (5)	0.58179 (11)	0.0277 (4)	
H18D	0.7715	0.6790	0.5795	0.042*	
H18E	0.9356	0.6832	0.6311	0.042*	
H18F	0.8985	0.6546	0.5674	0.042*	
C19B	1.3972 (2)	0.63691 (5)	0.62517 (11)	0.0271 (4)	
H19D	1.4889	0.6236	0.6429	0.041*	
H19E	1.3200	0.6211	0.5969	0.041*	
H19F	1.3698	0.6463	0.6660	0.041*	
C20B	1.4894 (2)	0.74387 (6)	0.45006 (13)	0.0386 (5)	
H20D	1.5918	0.7426	0.4510	0.058*	
H20E	1.4711	0.7669	0.4692	0.058*	
H20F	1.4258	0.7413	0.4004	0.058*	
C21B	0.4564 (2)	0.95365 (5)	0.28354 (11)	0.0254 (4)	
H21D	0.3852	0.9469	0.3080	0.038*	
H21E	0.4070	0.9671	0.2398	0.038*	
H21F	0.5326	0.9685	0.3157	0.038*	
C22B	0.7315 (2)	1.01884 (5)	0.13343 (12)	0.0280 (4)	
H22D	0.7693	1.0381	0.1103	0.042*	
H22E	0.7033	1.0284	0.1740	0.042*	
H22F	0.6461	1.0081	0.0985	0.042*	
C23B	1.1362 (2)	0.90947 (5)	0.19467 (11)	0.0255 (4)	
H23D	1.2174	0.9161	0.1768	0.038*	
H23E	1.0804	0.8901	0.1652	0.038*	
H23F	1.1747	0.9016	0.2447	0.038*	
O1C	0.17116 (13)	0.85328 (4)	0.35904 (7)	0.0250 (3)	
O2C	0.36440 (14)	0.94816 (3)	0.52907 (7)	0.0225 (3)	
O3C	0.88225 (14)	0.98683 (3)	0.59876 (7)	0.0230 (3)	
O4C	0.93952 (13)	0.93975 (3)	0.51207 (7)	0.0228 (3)	
O5C	0.00738 (14)	0.74097 (3)	0.20661 (7)	0.0238 (3)	
O6C	0.33553 (14)	0.68688 (3)	0.08487 (7)	0.0235 (3)	
O7C	0.54343 (14)	0.73320 (4)	0.14497 (8)	0.0269 (3)	
C1C	0.50778 (18)	0.94655 (5)	0.52632 (9)	0.0174 (3)	
C2C	0.62239 (19)	0.96902 (5)	0.56811 (9)	0.0182 (3)	
H2CA	0.6031	0.9860	0.6000	0.022*	
C3C	0.76404 (19)	0.96585 (5)	0.56170 (9)	0.0176 (3)	
C4C	0.79536 (18)	0.94012 (5)	0.51384 (10)	0.0178 (3)	
C5C	0.68220 (18)	0.91813 (5)	0.47306 (9)	0.0168 (3)	
H5CA	0.7024	0.9012	0.4413	0.020*	
C6C	0.53558 (18)	0.92067 (4)	0.47832 (9)	0.0163 (3)	
C7C	0.41633 (18)	0.89700 (4)	0.43687 (9)	0.0165 (3)	
H7CA	0.3206	0.9029	0.4374	0.020*	
C8C	0.42894 (18)	0.86764 (4)	0.39794 (9)	0.0164 (3)	
H8CA	0.5230	0.8606	0.3967	0.020*	
C9C	0.29764 (18)	0.84615 (4)	0.35702 (9)	0.0157 (3)	
C10C	0.32957 (18)	0.81622 (5)	0.31329 (9)	0.0178 (3)	
H10C	0.4278	0.8127	0.3141	0.021*	
C11C	0.22491 (18)	0.79370 (5)	0.27220 (9)	0.0168 (3)	
H11C	0.1277	0.7966	0.2735	0.020*	
C12C	0.25297 (18)	0.76502 (5)	0.22576 (9)	0.0167 (3)	
C13C	0.14380 (18)	0.73940 (5)	0.19315 (9)	0.0180 (3)	
C14C	0.16725 (19)	0.71267 (5)	0.14619 (10)	0.0197 (3)	
H14C	0.0928	0.6958	0.1254	0.024*	
C15C	0.30085 (19)	0.71122 (5)	0.13060 (10)	0.0191 (3)	
C16C	0.41492 (19)	0.73680 (5)	0.16373 (10)	0.0196 (4)	
C17C	0.38995 (19)	0.76270 (5)	0.20996 (10)	0.0196 (4)	
H17C	0.4652	0.7792	0.2316	0.023*	
C18C	0.3321 (2)	0.97249 (5)	0.58045 (10)	0.0225 (4)	
H18G	0.2274	0.9717	0.5750	0.034*	
H18H	0.3597	0.9967	0.5714	0.034*	
H18I	0.3876	0.9654	0.6295	0.034*	
C19C	0.8569 (2)	1.01450 (5)	0.64584 (11)	0.0271 (4)	
H19G	0.9477	1.0277	0.6674	0.041*	
H19H	0.8244	1.0036	0.6839	0.041*	
H19I	0.7815	1.0308	0.6178	0.041*	
C20C	0.9770 (2)	0.91258 (6)	0.46759 (12)	0.0299 (4)	
H20G	1.0814	0.9141	0.4724	0.045*	
H20H	0.9197	0.9163	0.4172	0.045*	
H20I	0.9552	0.8890	0.4832	0.045*	
C21C	−0.0131 (2)	0.71272 (6)	0.25332 (12)	0.0330 (5)	
H21G	−0.1110	0.7146	0.2587	0.049*	
H21H	0.0604	0.7149	0.3006	0.049*	
H21I	−0.0026	0.6896	0.2322	0.049*	
C22C	0.2219 (2)	0.66088 (5)	0.05013 (11)	0.0283 (4)	
H22G	0.2587	0.6449	0.0200	0.042*	
H22H	0.1352	0.6734	0.0202	0.042*	
H22I	0.1960	0.6471	0.0870	0.042*	
C23C	0.6505 (2)	0.76178 (5)	0.16901 (12)	0.0302 (5)	
H23G	0.7337	0.7574	0.1511	0.045*	
H23H	0.6843	0.7625	0.2217	0.045*	
H23I	0.6051	0.7845	0.1504	0.045*	

### Atomic displacement parameters (Å^2^)

**Table:**

	*U*^11^	*U*^22^	*U*^33^	*U*^12^	*U*^13^	*U*^23^
O1A	0.0184 (6)	0.0268 (7)	0.0349 (8)	−0.0002 (5)	0.0116 (5)	−0.0028 (6)
O2A	0.0277 (7)	0.0229 (7)	0.0320 (8)	0.0025 (5)	0.0152 (6)	−0.0038 (6)
O3A	0.0293 (7)	0.0241 (7)	0.0269 (7)	−0.0018 (5)	0.0033 (6)	−0.0076 (6)
O4A	0.0213 (7)	0.0316 (8)	0.0291 (7)	−0.0044 (5)	0.0072 (5)	−0.0085 (6)
O5A	0.0151 (6)	0.0236 (7)	0.0350 (8)	0.0000 (5)	0.0092 (5)	0.0000 (6)
O6A	0.0234 (7)	0.0235 (7)	0.0270 (7)	0.0004 (5)	0.0112 (5)	−0.0027 (5)
O7A	0.0188 (6)	0.0252 (7)	0.0376 (8)	−0.0012 (5)	0.0155 (6)	−0.0026 (6)
C1A	0.0244 (9)	0.0181 (9)	0.0196 (9)	0.0037 (7)	0.0084 (7)	0.0048 (7)
C2A	0.0314 (10)	0.0172 (9)	0.0183 (9)	0.0046 (7)	0.0069 (7)	0.0014 (7)
C3A	0.0266 (9)	0.0181 (9)	0.0170 (9)	−0.0005 (7)	0.0010 (7)	0.0021 (7)
C4A	0.0217 (9)	0.0228 (9)	0.0185 (9)	0.0005 (7)	0.0054 (7)	0.0019 (7)
C5A	0.0225 (9)	0.0186 (9)	0.0175 (9)	0.0019 (6)	0.0051 (7)	0.0018 (7)
C6A	0.0217 (9)	0.0173 (9)	0.0173 (8)	0.0018 (6)	0.0054 (7)	0.0027 (7)
C7A	0.0184 (8)	0.0195 (9)	0.0201 (9)	0.0019 (6)	0.0071 (7)	0.0049 (7)
C8A	0.0168 (8)	0.0174 (8)	0.0212 (9)	0.0015 (6)	0.0060 (6)	0.0036 (7)
C9A	0.0193 (8)	0.0154 (8)	0.0196 (9)	0.0027 (6)	0.0058 (6)	0.0057 (7)
C10A	0.0156 (8)	0.0211 (9)	0.0222 (9)	0.0021 (6)	0.0072 (6)	0.0029 (7)
C11A	0.0157 (8)	0.0190 (9)	0.0231 (9)	0.0030 (6)	0.0070 (6)	0.0044 (7)
C12A	0.0165 (8)	0.0181 (9)	0.0194 (9)	0.0017 (6)	0.0053 (6)	0.0036 (7)
C13A	0.0144 (8)	0.0190 (9)	0.0247 (9)	0.0018 (6)	0.0065 (6)	0.0042 (7)
C14A	0.0170 (8)	0.0200 (9)	0.0222 (9)	−0.0006 (6)	0.0045 (7)	0.0016 (7)
C15A	0.0211 (9)	0.0180 (9)	0.0188 (9)	0.0029 (6)	0.0069 (7)	0.0024 (7)
C16A	0.0151 (8)	0.0199 (9)	0.0234 (9)	0.0021 (6)	0.0076 (6)	0.0045 (7)
C17A	0.0158 (8)	0.0183 (9)	0.0227 (9)	−0.0005 (6)	0.0052 (6)	0.0021 (7)
C18A	0.0340 (11)	0.0280 (11)	0.0315 (11)	0.0058 (8)	0.0165 (9)	−0.0032 (9)
C19A	0.0364 (11)	0.0216 (10)	0.0291 (11)	0.0049 (8)	−0.0010 (8)	−0.0069 (8)
C20A	0.0237 (10)	0.0398 (12)	0.0347 (11)	−0.0057 (8)	0.0124 (8)	−0.0117 (9)
C21A	0.0184 (9)	0.0304 (11)	0.0297 (10)	0.0001 (7)	0.0089 (7)	0.0029 (8)
C22A	0.0307 (10)	0.0241 (10)	0.0277 (10)	−0.0030 (8)	0.0107 (8)	−0.0049 (8)
C23A	0.0157 (9)	0.0274 (10)	0.0399 (12)	0.0005 (7)	0.0110 (8)	0.0049 (9)
O1B	0.0167 (6)	0.0280 (7)	0.0447 (9)	0.0014 (5)	0.0103 (6)	0.0142 (6)
O2B	0.0206 (7)	0.0277 (7)	0.0363 (8)	0.0024 (5)	0.0125 (6)	0.0091 (6)
O3B	0.0198 (6)	0.0260 (7)	0.0323 (8)	0.0043 (5)	0.0049 (5)	0.0127 (6)
O4B	0.0185 (7)	0.0335 (8)	0.0384 (8)	0.0035 (5)	0.0105 (6)	0.0149 (6)
O5B	0.0175 (6)	0.0169 (6)	0.0377 (8)	0.0012 (5)	0.0159 (5)	0.0003 (5)
O6B	0.0194 (6)	0.0195 (7)	0.0342 (8)	0.0009 (5)	0.0114 (5)	0.0082 (6)
O7B	0.0169 (6)	0.0215 (7)	0.0284 (7)	0.0008 (5)	0.0113 (5)	0.0036 (5)
C1B	0.0194 (8)	0.0194 (9)	0.0216 (9)	−0.0014 (6)	0.0064 (7)	−0.0017 (7)
C2B	0.0233 (9)	0.0165 (9)	0.0192 (9)	−0.0012 (6)	0.0042 (7)	0.0016 (7)
C3B	0.0201 (9)	0.0187 (9)	0.0202 (9)	0.0022 (6)	0.0009 (7)	0.0014 (7)
C4B	0.0174 (8)	0.0238 (9)	0.0237 (9)	0.0004 (7)	0.0047 (7)	0.0017 (7)
C5B	0.0208 (9)	0.0182 (9)	0.0213 (9)	−0.0011 (6)	0.0051 (7)	0.0028 (7)
C6B	0.0188 (8)	0.0168 (9)	0.0198 (9)	0.0007 (6)	0.0029 (6)	−0.0008 (7)
C7B	0.0158 (8)	0.0197 (9)	0.0227 (9)	−0.0006 (6)	0.0051 (6)	−0.0017 (7)
C8B	0.0150 (8)	0.0198 (9)	0.0251 (9)	0.0013 (6)	0.0056 (7)	0.0012 (7)
C9B	0.0154 (8)	0.0161 (8)	0.0219 (9)	0.0005 (6)	0.0034 (6)	0.0001 (7)
C10B	0.0118 (8)	0.0207 (9)	0.0268 (9)	−0.0015 (6)	0.0055 (6)	0.0008 (7)
C11B	0.0131 (8)	0.0178 (8)	0.0204 (9)	−0.0025 (6)	0.0054 (6)	−0.0011 (7)
C12B	0.0143 (8)	0.0151 (8)	0.0155 (8)	−0.0016 (6)	0.0025 (6)	−0.0013 (6)
C13B	0.0126 (7)	0.0169 (8)	0.0195 (8)	−0.0030 (6)	0.0047 (6)	−0.0023 (7)
C14B	0.0169 (8)	0.0145 (8)	0.0224 (9)	0.0011 (6)	0.0044 (6)	−0.0014 (7)
C15B	0.0179 (8)	0.0143 (8)	0.0179 (8)	−0.0024 (6)	0.0039 (6)	−0.0004 (6)
C16B	0.0132 (8)	0.0205 (9)	0.0165 (8)	−0.0018 (6)	0.0040 (6)	−0.0021 (7)
C17B	0.0139 (8)	0.0177 (8)	0.0189 (8)	0.0010 (6)	0.0035 (6)	−0.0009 (7)
C18B	0.0292 (10)	0.0247 (10)	0.0336 (11)	−0.0028 (8)	0.0159 (8)	0.0034 (8)
C19B	0.0267 (10)	0.0226 (10)	0.0291 (10)	0.0047 (7)	0.0042 (8)	0.0104 (8)
C20B	0.0231 (10)	0.0437 (13)	0.0514 (14)	0.0011 (9)	0.0148 (9)	0.0236 (11)
C21B	0.0282 (10)	0.0212 (10)	0.0328 (11)	0.0048 (7)	0.0182 (8)	0.0007 (8)
C22B	0.0249 (10)	0.0202 (10)	0.0412 (12)	0.0035 (7)	0.0138 (8)	0.0092 (8)
C23B	0.0186 (9)	0.0259 (10)	0.0354 (11)	0.0047 (7)	0.0135 (8)	0.0057 (8)
O1C	0.0131 (6)	0.0277 (7)	0.0340 (8)	−0.0007 (5)	0.0070 (5)	−0.0082 (6)
O2C	0.0175 (6)	0.0241 (7)	0.0276 (7)	−0.0011 (5)	0.0093 (5)	−0.0071 (5)
O3C	0.0173 (6)	0.0227 (7)	0.0262 (7)	−0.0032 (5)	0.0026 (5)	−0.0091 (5)
O4C	0.0134 (6)	0.0262 (7)	0.0285 (7)	−0.0025 (5)	0.0056 (5)	−0.0082 (5)
O5C	0.0167 (6)	0.0236 (7)	0.0338 (7)	−0.0039 (5)	0.0118 (5)	−0.0013 (6)
O6C	0.0228 (7)	0.0217 (7)	0.0272 (7)	−0.0021 (5)	0.0096 (5)	−0.0085 (5)
O7C	0.0189 (6)	0.0256 (7)	0.0410 (8)	−0.0032 (5)	0.0162 (6)	−0.0091 (6)
C1C	0.0152 (8)	0.0168 (8)	0.0204 (9)	0.0004 (6)	0.0058 (6)	0.0021 (7)
C2C	0.0189 (8)	0.0157 (8)	0.0187 (8)	0.0002 (6)	0.0039 (6)	−0.0011 (7)
C3C	0.0181 (8)	0.0159 (8)	0.0159 (8)	−0.0013 (6)	0.0010 (6)	−0.0001 (7)
C4C	0.0139 (8)	0.0181 (9)	0.0203 (9)	0.0001 (6)	0.0039 (6)	0.0005 (7)
C5C	0.0148 (8)	0.0172 (8)	0.0176 (8)	0.0000 (6)	0.0036 (6)	−0.0006 (7)
C6C	0.0156 (8)	0.0149 (8)	0.0168 (8)	−0.0009 (6)	0.0025 (6)	0.0011 (6)
C7C	0.0137 (7)	0.0168 (8)	0.0186 (8)	−0.0001 (6)	0.0043 (6)	0.0035 (7)
C8C	0.0118 (7)	0.0169 (8)	0.0199 (8)	−0.0004 (6)	0.0038 (6)	0.0002 (7)
C9C	0.0149 (8)	0.0140 (8)	0.0173 (8)	−0.0008 (6)	0.0036 (6)	0.0017 (6)
C10C	0.0135 (8)	0.0174 (8)	0.0230 (9)	0.0009 (6)	0.0062 (6)	0.0003 (7)
C11C	0.0138 (8)	0.0171 (8)	0.0202 (9)	0.0007 (6)	0.0060 (6)	0.0017 (7)
C12C	0.0161 (8)	0.0158 (8)	0.0183 (8)	−0.0006 (6)	0.0054 (6)	0.0009 (7)
C13C	0.0142 (8)	0.0191 (9)	0.0210 (9)	0.0002 (6)	0.0058 (6)	0.0021 (7)
C14C	0.0180 (8)	0.0167 (9)	0.0227 (9)	−0.0037 (6)	0.0035 (7)	−0.0018 (7)
C15C	0.0203 (8)	0.0175 (9)	0.0197 (9)	0.0002 (6)	0.0063 (7)	−0.0009 (7)
C16C	0.0147 (8)	0.0199 (9)	0.0256 (9)	0.0003 (6)	0.0081 (7)	0.0003 (7)
C17C	0.0154 (8)	0.0188 (9)	0.0243 (9)	−0.0035 (6)	0.0057 (7)	−0.0028 (7)
C18C	0.0220 (9)	0.0237 (10)	0.0241 (9)	0.0004 (7)	0.0107 (7)	−0.0035 (8)
C19C	0.0246 (10)	0.0252 (10)	0.0281 (10)	−0.0024 (7)	0.0028 (8)	−0.0109 (8)
C20C	0.0170 (9)	0.0371 (12)	0.0367 (11)	−0.0013 (8)	0.0096 (8)	−0.0126 (9)
C21C	0.0330 (11)	0.0345 (12)	0.0387 (12)	−0.0051 (9)	0.0217 (9)	0.0023 (9)
C22C	0.0293 (10)	0.0265 (10)	0.0285 (10)	−0.0045 (8)	0.0079 (8)	−0.0109 (8)
C23C	0.0166 (9)	0.0306 (11)	0.0469 (13)	−0.0052 (7)	0.0150 (8)	−0.0111 (9)

### Geometric parameters (Å, °)

**Table:**

O1A—C9A	1.230 (2)	C11B—C12B	1.461 (2)
O2A—C1A	1.372 (2)	C11B—H11B	0.9300
O2A—C18A	1.435 (2)	C12B—C13B	1.401 (2)
O3A—C3A	1.363 (2)	C12B—C17B	1.407 (2)
O3A—C19A	1.427 (2)	C13B—C14B	1.399 (2)
O4A—C4A	1.367 (2)	C14B—C15B	1.385 (2)
O4A—C20A	1.426 (2)	C14B—H14B	0.9300
O5A—C13A	1.386 (2)	C15B—C16B	1.415 (2)
O5A—C21A	1.433 (2)	C16B—C17B	1.371 (2)
O6A—C15A	1.363 (2)	C17B—H17B	0.9300
O6A—C22A	1.433 (2)	C18B—H18D	0.9600
O7A—C16A	1.373 (2)	C18B—H18E	0.9600
O7A—C23A	1.425 (2)	C18B—H18F	0.9600
C1A—C2A	1.396 (3)	C19B—H19D	0.9600
C1A—C6A	1.403 (2)	C19B—H19E	0.9600
C2A—C3A	1.386 (3)	C19B—H19F	0.9600
C2A—H2AA	0.9300	C20B—H20D	0.9600
C3A—C4A	1.413 (3)	C20B—H20E	0.9600
C4A—C5A	1.376 (2)	C20B—H20F	0.9600
C5A—C6A	1.415 (2)	C21B—H21D	0.9600
C5A—H5AA	0.9300	C21B—H21E	0.9600
C6A—C7A	1.460 (2)	C21B—H21F	0.9600
C7A—C8A	1.343 (2)	C22B—H22D	0.9600
C7A—H7AA	0.9300	C22B—H22E	0.9600
C8A—C9A	1.472 (2)	C22B—H22F	0.9600
C8A—H8AA	0.9300	C23B—H23D	0.9600
C9A—C10A	1.479 (2)	C23B—H23E	0.9600
C10A—C11A	1.340 (2)	C23B—H23F	0.9600
C10A—H10A	0.9300	O1C—C9C	1.232 (2)
C11A—C12A	1.462 (2)	O2C—C1C	1.368 (2)
C11A—H11A	0.9300	O2C—C18C	1.430 (2)
C12A—C13A	1.394 (2)	O3C—C3C	1.363 (2)
C12A—C17A	1.417 (2)	O3C—C19C	1.428 (2)
C13A—C14A	1.400 (3)	O4C—C4C	1.368 (2)
C14A—C15A	1.385 (2)	O4C—C20C	1.426 (2)
C14A—H14A	0.9300	O5C—C13C	1.386 (2)
C15A—C16A	1.414 (2)	O5C—C21C	1.423 (2)
C16A—C17A	1.371 (2)	O6C—C15C	1.360 (2)
C17A—H17A	0.9300	O6C—C22C	1.439 (2)
C18A—H18A	0.9600	O7C—C16C	1.369 (2)
C18A—H18B	0.9600	O7C—C23C	1.433 (2)
C18A—H18C	0.9600	C1C—C2C	1.401 (2)
C19A—H19A	0.9600	C1C—C6C	1.403 (2)
C19A—H19B	0.9600	C2C—C3C	1.381 (2)
C19A—H19C	0.9600	C2C—H2CA	0.9300
C20A—H20A	0.9600	C3C—C4C	1.410 (2)
C20A—H20B	0.9600	C4C—C5C	1.376 (2)
C20A—H20C	0.9600	C5C—C6C	1.417 (2)
C21A—H21A	0.9600	C5C—H5CA	0.9300
C21A—H21B	0.9600	C6C—C7C	1.452 (2)
C21A—H21C	0.9600	C7C—C8C	1.339 (2)
C22A—H22A	0.9600	C7C—H7CA	0.9300
C22A—H22B	0.9600	C8C—C9C	1.476 (2)
C22A—H22C	0.9600	C8C—H8CA	0.9300
C23A—H23A	0.9600	C9C—C10C	1.470 (2)
C23A—H23B	0.9600	C10C—C11C	1.344 (2)
C23A—H23C	0.9600	C10C—H10C	0.9300
O1B—C9B	1.230 (2)	C11C—C12C	1.456 (2)
O2B—C1B	1.365 (2)	C11C—H11C	0.9300
O2B—C18B	1.428 (2)	C12C—C13C	1.395 (2)
O3B—C3B	1.366 (2)	C12C—C17C	1.414 (2)
O3B—C19B	1.425 (2)	C13C—C14C	1.395 (2)
O4B—C4B	1.366 (2)	C14C—C15C	1.379 (2)
O4B—C20B	1.428 (2)	C14C—H14C	0.9300
O5B—C13B	1.374 (2)	C15C—C16C	1.425 (2)
O5B—C21B	1.419 (2)	C16C—C17C	1.369 (2)
O6B—C15B	1.364 (2)	C17C—H17C	0.9300
O6B—C22B	1.435 (2)	C18C—H18G	0.9600
O7B—C16B	1.370 (2)	C18C—H18H	0.9600
O7B—C23B	1.425 (2)	C18C—H18I	0.9600
C1B—C6B	1.398 (2)	C19C—H19G	0.9600
C1B—C2B	1.404 (2)	C19C—H19H	0.9600
C2B—C3B	1.381 (3)	C19C—H19I	0.9600
C2B—H2BA	0.9300	C20C—H20G	0.9600
C3B—C4B	1.408 (3)	C20C—H20H	0.9600
C4B—C5B	1.379 (2)	C20C—H20I	0.9600
C5B—C6B	1.411 (2)	C21C—H21G	0.9600
C5B—H5BA	0.9300	C21C—H21H	0.9600
C6B—C7B	1.459 (2)	C21C—H21I	0.9600
C7B—C8B	1.337 (3)	C22C—H22G	0.9600
C7B—H7BA	0.9300	C22C—H22H	0.9600
C8B—C9B	1.477 (2)	C22C—H22I	0.9600
C8B—H8BA	0.9300	C23C—H23G	0.9600
C9B—C10B	1.470 (2)	C23C—H23H	0.9600
C10B—C11B	1.338 (2)	C23C—H23I	0.9600
C10B—H10B	0.9300		
			
C1A—O2A—C18A	118.38 (15)	C13B—C14B—H14B	120.1
C3A—O3A—C19A	117.03 (16)	O6B—C15B—C14B	124.72 (15)
C4A—O4A—C20A	116.68 (15)	O6B—C15B—C16B	115.41 (15)
C13A—O5A—C21A	114.34 (14)	C14B—C15B—C16B	119.86 (15)
C15A—O6A—C22A	116.90 (14)	O7B—C16B—C17B	124.78 (15)
C16A—O7A—C23A	116.30 (14)	O7B—C16B—C15B	115.77 (15)
O2A—C1A—C2A	122.55 (16)	C17B—C16B—C15B	119.45 (15)
O2A—C1A—C6A	116.50 (16)	C16B—C17B—C12B	121.93 (16)
C2A—C1A—C6A	120.95 (17)	C16B—C17B—H17B	119.0
C3A—C2A—C1A	120.04 (17)	C12B—C17B—H17B	119.0
C3A—C2A—H2AA	120.0	O2B—C18B—H18D	109.5
C1A—C2A—H2AA	120.0	O2B—C18B—H18E	109.5
O3A—C3A—C2A	124.48 (17)	H18D—C18B—H18E	109.5
O3A—C3A—C4A	115.16 (17)	O2B—C18B—H18F	109.5
C2A—C3A—C4A	120.34 (17)	H18D—C18B—H18F	109.5
O4A—C4A—C5A	126.01 (17)	H18E—C18B—H18F	109.5
O4A—C4A—C3A	115.09 (16)	O3B—C19B—H19D	109.5
C5A—C4A—C3A	118.90 (17)	O3B—C19B—H19E	109.5
C4A—C5A—C6A	122.12 (17)	H19D—C19B—H19E	109.5
C4A—C5A—H5AA	118.9	O3B—C19B—H19F	109.5
C6A—C5A—H5AA	118.9	H19D—C19B—H19F	109.5
C1A—C6A—C5A	117.64 (16)	H19E—C19B—H19F	109.5
C1A—C6A—C7A	120.82 (16)	O4B—C20B—H20D	109.5
C5A—C6A—C7A	121.53 (16)	O4B—C20B—H20E	109.5
C8A—C7A—C6A	125.57 (16)	H20D—C20B—H20E	109.5
C8A—C7A—H7AA	117.2	O4B—C20B—H20F	109.5
C6A—C7A—H7AA	117.2	H20D—C20B—H20F	109.5
C7A—C8A—C9A	122.90 (16)	H20E—C20B—H20F	109.5
C7A—C8A—H8AA	118.6	O5B—C21B—H21D	109.5
C9A—C8A—H8AA	118.6	O5B—C21B—H21E	109.5
O1A—C9A—C8A	123.11 (16)	H21D—C21B—H21E	109.5
O1A—C9A—C10A	122.22 (16)	O5B—C21B—H21F	109.5
C8A—C9A—C10A	114.65 (15)	H21D—C21B—H21F	109.5
C11A—C10A—C9A	122.98 (16)	H21E—C21B—H21F	109.5
C11A—C10A—H10A	118.5	O6B—C22B—H22D	109.5
C9A—C10A—H10A	118.5	O6B—C22B—H22E	109.5
C10A—C11A—C12A	126.25 (16)	H22D—C22B—H22E	109.5
C10A—C11A—H11A	116.9	O6B—C22B—H22F	109.5
C12A—C11A—H11A	116.9	H22D—C22B—H22F	109.5
C13A—C12A—C17A	117.40 (16)	H22E—C22B—H22F	109.5
C13A—C12A—C11A	120.89 (16)	O7B—C23B—H23D	109.5
C17A—C12A—C11A	121.71 (16)	O7B—C23B—H23E	109.5
O5A—C13A—C12A	119.63 (16)	H23D—C23B—H23E	109.5
O5A—C13A—C14A	118.70 (16)	O7B—C23B—H23F	109.5
C12A—C13A—C14A	121.61 (16)	H23D—C23B—H23F	109.5
C15A—C14A—C13A	119.79 (16)	H23E—C23B—H23F	109.5
C15A—C14A—H14A	120.1	C1C—O2C—C18C	118.19 (14)
C13A—C14A—H14A	120.1	C3C—O3C—C19C	117.77 (14)
O6A—C15A—C14A	124.94 (16)	C4C—O4C—C20C	116.72 (14)
O6A—C15A—C16A	115.38 (15)	C13C—O5C—C21C	113.38 (14)
C14A—C15A—C16A	119.68 (16)	C15C—O6C—C22C	116.48 (14)
C17A—C16A—O7A	125.07 (16)	C16C—O7C—C23C	115.83 (14)
C17A—C16A—C15A	119.80 (16)	O2C—C1C—C2C	123.35 (15)
O7A—C16A—C15A	115.13 (15)	O2C—C1C—C6C	115.95 (15)
C16A—C17A—C12A	121.71 (16)	C2C—C1C—C6C	120.70 (15)
C16A—C17A—H17A	119.1	C3C—C2C—C1C	119.73 (16)
C12A—C17A—H17A	119.1	C3C—C2C—H2CA	120.1
O2A—C18A—H18A	109.5	C1C—C2C—H2CA	120.1
O2A—C18A—H18B	109.5	O3C—C3C—C2C	124.29 (16)
H18A—C18A—H18B	109.5	O3C—C3C—C4C	114.83 (15)
O2A—C18A—H18C	109.5	C2C—C3C—C4C	120.88 (15)
H18A—C18A—H18C	109.5	O4C—C4C—C5C	125.64 (16)
H18B—C18A—H18C	109.5	O4C—C4C—C3C	115.35 (15)
O3A—C19A—H19A	109.5	C5C—C4C—C3C	119.01 (16)
O3A—C19A—H19B	109.5	C4C—C5C—C6C	121.57 (16)
H19A—C19A—H19B	109.5	C4C—C5C—H5CA	119.2
O3A—C19A—H19C	109.5	C6C—C5C—H5CA	119.2
H19A—C19A—H19C	109.5	C1C—C6C—C5C	118.11 (15)
H19B—C19A—H19C	109.5	C1C—C6C—C7C	119.90 (15)
O4A—C20A—H20A	109.5	C5C—C6C—C7C	121.98 (15)
O4A—C20A—H20B	109.5	C8C—C7C—C6C	127.18 (16)
H20A—C20A—H20B	109.5	C8C—C7C—H7CA	116.4
O4A—C20A—H20C	109.5	C6C—C7C—H7CA	116.4
H20A—C20A—H20C	109.5	C7C—C8C—C9C	121.77 (15)
H20B—C20A—H20C	109.5	C7C—C8C—H8CA	119.1
O5A—C21A—H21A	109.5	C9C—C8C—H8CA	119.1
O5A—C21A—H21B	109.5	O1C—C9C—C10C	123.05 (15)
H21A—C21A—H21B	109.5	O1C—C9C—C8C	122.05 (15)
O5A—C21A—H21C	109.5	C10C—C9C—C8C	114.90 (14)
H21A—C21A—H21C	109.5	C11C—C10C—C9C	123.76 (15)
H21B—C21A—H21C	109.5	C11C—C10C—H10C	118.1
O6A—C22A—H22A	109.5	C9C—C10C—H10C	118.1
O6A—C22A—H22B	109.5	C10C—C11C—C12C	124.63 (15)
H22A—C22A—H22B	109.5	C10C—C11C—H11C	117.7
O6A—C22A—H22C	109.5	C12C—C11C—H11C	117.7
H22A—C22A—H22C	109.5	C13C—C12C—C17C	117.36 (15)
H22B—C22A—H22C	109.5	C13C—C12C—C11C	121.21 (15)
O7A—C23A—H23A	109.5	C17C—C12C—C11C	121.40 (15)
O7A—C23A—H23B	109.5	O5C—C13C—C12C	119.15 (15)
H23A—C23A—H23B	109.5	O5C—C13C—C14C	119.10 (15)
O7A—C23A—H23C	109.5	C12C—C13C—C14C	121.74 (15)
H23A—C23A—H23C	109.5	C15C—C14C—C13C	120.00 (16)
H23B—C23A—H23C	109.5	C15C—C14C—H14C	120.0
C1B—O2B—C18B	119.11 (14)	C13C—C14C—H14C	120.0
C3B—O3B—C19B	117.48 (15)	O6C—C15C—C14C	125.29 (16)
C4B—O4B—C20B	116.76 (15)	O6C—C15C—C16C	115.24 (15)
C13B—O5B—C21B	116.60 (13)	C14C—C15C—C16C	119.47 (16)
C15B—O6B—C22B	116.72 (14)	C17C—C16C—O7C	125.18 (16)
C16B—O7B—C23B	115.94 (14)	C17C—C16C—C15C	119.61 (16)
O2B—C1B—C6B	116.44 (15)	O7C—C16C—C15C	115.21 (15)
O2B—C1B—C2B	123.29 (16)	C16C—C17C—C12C	121.81 (16)
C6B—C1B—C2B	120.27 (16)	C16C—C17C—H17C	119.1
C3B—C2B—C1B	119.69 (16)	C12C—C17C—H17C	119.1
C3B—C2B—H2BA	120.2	O2C—C18C—H18G	109.5
C1B—C2B—H2BA	120.2	O2C—C18C—H18H	109.5
O3B—C3B—C2B	124.24 (16)	H18G—C18C—H18H	109.5
O3B—C3B—C4B	114.66 (16)	O2C—C18C—H18I	109.5
C2B—C3B—C4B	121.10 (16)	H18G—C18C—H18I	109.5
O4B—C4B—C5B	125.85 (17)	H18H—C18C—H18I	109.5
O4B—C4B—C3B	115.39 (16)	O3C—C19C—H19G	109.5
C5B—C4B—C3B	118.76 (17)	O3C—C19C—H19H	109.5
C4B—C5B—C6B	121.41 (17)	H19G—C19C—H19H	109.5
C4B—C5B—H5BA	119.3	O3C—C19C—H19I	109.5
C6B—C5B—H5BA	119.3	H19G—C19C—H19I	109.5
C1B—C6B—C5B	118.77 (16)	H19H—C19C—H19I	109.5
C1B—C6B—C7B	119.06 (16)	O4C—C20C—H20G	109.5
C5B—C6B—C7B	122.17 (16)	O4C—C20C—H20H	109.5
C8B—C7B—C6B	127.45 (16)	H20G—C20C—H20H	109.5
C8B—C7B—H7BA	116.3	O4C—C20C—H20I	109.5
C6B—C7B—H7BA	116.3	H20G—C20C—H20I	109.5
C7B—C8B—C9B	121.37 (16)	H20H—C20C—H20I	109.5
C7B—C8B—H8BA	119.3	O5C—C21C—H21G	109.5
C9B—C8B—H8BA	119.3	O5C—C21C—H21H	109.5
O1B—C9B—C10B	122.79 (15)	H21G—C21C—H21H	109.5
O1B—C9B—C8B	123.08 (16)	O5C—C21C—H21I	109.5
C10B—C9B—C8B	114.11 (15)	H21G—C21C—H21I	109.5
C11B—C10B—C9B	124.36 (16)	H21H—C21C—H21I	109.5
C11B—C10B—H10B	117.8	O6C—C22C—H22G	109.5
C9B—C10B—H10B	117.8	O6C—C22C—H22H	109.5
C10B—C11B—C12B	124.00 (15)	H22G—C22C—H22H	109.5
C10B—C11B—H11B	118.0	O6C—C22C—H22I	109.5
C12B—C11B—H11B	118.0	H22G—C22C—H22I	109.5
C13B—C12B—C17B	117.80 (15)	H22H—C22C—H22I	109.5
C13B—C12B—C11B	121.06 (15)	O7C—C23C—H23G	109.5
C17B—C12B—C11B	121.12 (15)	O7C—C23C—H23H	109.5
O5B—C13B—C14B	122.15 (15)	H23G—C23C—H23H	109.5
O5B—C13B—C12B	116.78 (14)	O7C—C23C—H23I	109.5
C14B—C13B—C12B	121.07 (15)	H23G—C23C—H23I	109.5
C15B—C14B—C13B	119.85 (15)	H23H—C23C—H23I	109.5
C15B—C14B—H14B	120.1		
			
C18A—O2A—C1A—C2A	−3.7 (2)	O1B—C9B—C10B—C11B	5.5 (3)
C18A—O2A—C1A—C6A	177.34 (16)	C8B—C9B—C10B—C11B	−173.08 (17)
O2A—C1A—C2A—C3A	−178.51 (16)	C9B—C10B—C11B—C12B	176.23 (16)
C6A—C1A—C2A—C3A	0.4 (3)	C10B—C11B—C12B—C13B	−166.56 (17)
C19A—O3A—C3A—C2A	−0.2 (3)	C10B—C11B—C12B—C17B	11.8 (3)
C19A—O3A—C3A—C4A	178.80 (16)	C21B—O5B—C13B—C14B	−30.2 (2)
C1A—C2A—C3A—O3A	178.18 (16)	C21B—O5B—C13B—C12B	150.82 (16)
C1A—C2A—C3A—C4A	−0.8 (3)	C17B—C12B—C13B—O5B	−179.65 (15)
C20A—O4A—C4A—C5A	3.8 (3)	C11B—C12B—C13B—O5B	−1.3 (2)
C20A—O4A—C4A—C3A	−176.10 (17)	C17B—C12B—C13B—C14B	1.4 (2)
O3A—C3A—C4A—O4A	1.9 (2)	C11B—C12B—C13B—C14B	179.73 (16)
C2A—C3A—C4A—O4A	−179.01 (16)	O5B—C13B—C14B—C15B	179.36 (15)
O3A—C3A—C4A—C5A	−178.01 (16)	C12B—C13B—C14B—C15B	−1.7 (3)
C2A—C3A—C4A—C5A	1.1 (3)	C22B—O6B—C15B—C14B	−7.6 (3)
O4A—C4A—C5A—C6A	179.13 (17)	C22B—O6B—C15B—C16B	171.36 (16)
C3A—C4A—C5A—C6A	−1.0 (3)	C13B—C14B—C15B—O6B	179.04 (16)
O2A—C1A—C6A—C5A	178.71 (15)	C13B—C14B—C15B—C16B	0.2 (3)
C2A—C1A—C6A—C5A	−0.3 (3)	C23B—O7B—C16B—C17B	9.6 (2)
O2A—C1A—C6A—C7A	−0.8 (2)	C23B—O7B—C16B—C15B	−169.73 (15)
C2A—C1A—C6A—C7A	−179.84 (16)	O6B—C15B—C16B—O7B	2.1 (2)
C4A—C5A—C6A—C1A	0.6 (3)	C14B—C15B—C16B—O7B	−178.93 (15)
C4A—C5A—C6A—C7A	−179.88 (16)	O6B—C15B—C16B—C17B	−177.28 (15)
C1A—C6A—C7A—C8A	−177.55 (17)	C14B—C15B—C16B—C17B	1.7 (2)
C5A—C6A—C7A—C8A	2.9 (3)	O7B—C16B—C17B—C12B	178.63 (15)
C6A—C7A—C8A—C9A	−175.45 (16)	C15B—C16B—C17B—C12B	−2.1 (3)
C7A—C8A—C9A—O1A	−8.2 (3)	C13B—C12B—C17B—C16B	0.6 (2)
C7A—C8A—C9A—C10A	170.30 (16)	C11B—C12B—C17B—C16B	−177.81 (16)
O1A—C9A—C10A—C11A	−4.0 (3)	C18C—O2C—C1C—C2C	−3.9 (2)
C8A—C9A—C10A—C11A	177.49 (16)	C18C—O2C—C1C—C6C	176.17 (15)
C9A—C10A—C11A—C12A	−179.03 (16)	O2C—C1C—C2C—C3C	−179.51 (16)
C10A—C11A—C12A—C13A	−179.58 (17)	C6C—C1C—C2C—C3C	0.4 (3)
C10A—C11A—C12A—C17A	0.4 (3)	C19C—O3C—C3C—C2C	−1.7 (2)
C21A—O5A—C13A—C12A	−111.48 (18)	C19C—O3C—C3C—C4C	177.40 (16)
C21A—O5A—C13A—C14A	71.4 (2)	C1C—C2C—C3C—O3C	178.69 (16)
C17A—C12A—C13A—O5A	−175.63 (15)	C1C—C2C—C3C—C4C	−0.3 (3)
C11A—C12A—C13A—O5A	4.3 (2)	C20C—O4C—C4C—C5C	−4.3 (3)
C17A—C12A—C13A—C14A	1.4 (3)	C20C—O4C—C4C—C3C	176.31 (16)
C11A—C12A—C13A—C14A	−178.64 (16)	O3C—C3C—C4C—O4C	0.6 (2)
O5A—C13A—C14A—C15A	176.04 (16)	C2C—C3C—C4C—O4C	179.66 (15)
C12A—C13A—C14A—C15A	−1.0 (3)	O3C—C3C—C4C—C5C	−178.86 (15)
C22A—O6A—C15A—C14A	1.1 (3)	C2C—C3C—C4C—C5C	0.2 (3)
C22A—O6A—C15A—C16A	−177.98 (15)	O4C—C4C—C5C—C6C	−179.61 (16)
C13A—C14A—C15A—O6A	−179.14 (16)	C3C—C4C—C5C—C6C	−0.2 (3)
C13A—C14A—C15A—C16A	−0.1 (3)	O2C—C1C—C6C—C5C	179.51 (15)
C23A—O7A—C16A—C17A	−14.4 (3)	C2C—C1C—C6C—C5C	−0.4 (2)
C23A—O7A—C16A—C15A	164.98 (16)	O2C—C1C—C6C—C7C	−1.6 (2)
O6A—C15A—C16A—C17A	179.90 (15)	C2C—C1C—C6C—C7C	178.44 (16)
C14A—C15A—C16A—C17A	0.8 (3)	C4C—C5C—C6C—C1C	0.3 (3)
O6A—C15A—C16A—O7A	0.5 (2)	C4C—C5C—C6C—C7C	−178.49 (16)
C14A—C15A—C16A—O7A	−178.62 (16)	C1C—C6C—C7C—C8C	−168.99 (17)
O7A—C16A—C17A—C12A	178.97 (16)	C5C—C6C—C7C—C8C	9.8 (3)
C15A—C16A—C17A—C12A	−0.3 (3)	C6C—C7C—C8C—C9C	−178.94 (16)
C13A—C12A—C17A—C16A	−0.7 (3)	C7C—C8C—C9C—O1C	−3.0 (3)
C11A—C12A—C17A—C16A	179.33 (16)	C7C—C8C—C9C—C10C	176.17 (16)
C18B—O2B—C1B—C6B	−179.45 (16)	O1C—C9C—C10C—C11C	−0.7 (3)
C18B—O2B—C1B—C2B	1.4 (3)	C8C—C9C—C10C—C11C	−179.82 (16)
O2B—C1B—C2B—C3B	179.33 (17)	C9C—C10C—C11C—C12C	176.90 (16)
C6B—C1B—C2B—C3B	0.2 (3)	C10C—C11C—C12C—C13C	170.52 (17)
C19B—O3B—C3B—C2B	3.5 (3)	C10C—C11C—C12C—C17C	−11.6 (3)
C19B—O3B—C3B—C4B	−176.27 (16)	C21C—O5C—C13C—C12C	−107.71 (19)
C1B—C2B—C3B—O3B	−179.63 (17)	C21C—O5C—C13C—C14C	73.5 (2)
C1B—C2B—C3B—C4B	0.2 (3)	C17C—C12C—C13C—O5C	−179.32 (15)
C20B—O4B—C4B—C5B	4.4 (3)	C11C—C12C—C13C—O5C	−1.3 (2)
C20B—O4B—C4B—C3B	−176.09 (18)	C17C—C12C—C13C—C14C	−0.6 (3)
O3B—C3B—C4B—O4B	−0.2 (2)	C11C—C12C—C13C—C14C	177.38 (16)
C2B—C3B—C4B—O4B	179.97 (17)	O5C—C13C—C14C—C15C	178.35 (16)
O3B—C3B—C4B—C5B	179.36 (16)	C12C—C13C—C14C—C15C	−0.4 (3)
C2B—C3B—C4B—C5B	−0.5 (3)	C22C—O6C—C15C—C14C	0.4 (3)
O4B—C4B—C5B—C6B	179.85 (17)	C22C—O6C—C15C—C16C	−179.51 (16)
C3B—C4B—C5B—C6B	0.3 (3)	C13C—C14C—C15C—O6C	−178.86 (16)
O2B—C1B—C6B—C5B	−179.52 (16)	C13C—C14C—C15C—C16C	1.0 (3)
C2B—C1B—C6B—C5B	−0.4 (3)	C23C—O7C—C16C—C17C	−8.2 (3)
O2B—C1B—C6B—C7B	0.1 (2)	C23C—O7C—C16C—C15C	171.17 (17)
C2B—C1B—C6B—C7B	179.22 (16)	O6C—C15C—C16C—C17C	179.22 (16)
C4B—C5B—C6B—C1B	0.1 (3)	C14C—C15C—C16C—C17C	−0.7 (3)
C4B—C5B—C6B—C7B	−179.51 (17)	O6C—C15C—C16C—O7C	−0.2 (2)
C1B—C6B—C7B—C8B	176.72 (18)	C14C—C15C—C16C—O7C	179.96 (16)
C5B—C6B—C7B—C8B	−3.7 (3)	O7C—C16C—C17C—C12C	178.97 (17)
C6B—C7B—C8B—C9B	−179.50 (17)	C15C—C16C—C17C—C12C	−0.3 (3)
C7B—C8B—C9B—O1B	−6.0 (3)	C13C—C12C—C17C—C16C	1.0 (3)
C7B—C8B—C9B—C10B	172.61 (17)	C11C—C12C—C17C—C16C	−177.03 (17)

### Hydrogen-bond geometry (Å, °)

**Table:**

Cg3 and Cg4 are the centroids of the C1C–C6C and C12C–C17C rings, respectively.

**Table:**

*D*—H···*A*	*D*—H	H···*A*	*D*···*A*	*D*—H···*A*
C10B—H10B···O1C^i^	0.93	2.29	3.149 (2)	153
C10C—H10C···O1B	0.93	2.33	3.195 (2)	155
C14B—H14B···O1A^ii^	0.93	2.52	3.353 (2)	149
C21A—H21B···O3A^iii^	0.96	2.49	3.301 (2)	142
C22A—H22C···O7C^iv^	0.96	2.50	3.435 (2)	165
C22B—H22D···O2A^v^	0.96	2.50	3.407 (2)	158
C22B—H22F···O1A^ii^	0.96	2.58	3.227 (2)	125
C23A—H23A···O5A^i^	0.96	2.52	3.308 (2)	140
C23A—H23C···O3C	0.96	2.53	3.452 (2)	161
C23C—H23G···O5C^i^	0.96	2.54	3.305 (2)	136
C18C—H18H···Cg4^ii^	0.96	2.80	3.678 (2)	152
C20A—H20B···Cg3^vi^	0.96	2.94	3.855 (2)	159

Symmetry codes: (i) *x*+1, *y*, *z*;  (ii) −*x*+1, −*y*+2, −*z*+1; (iii) −*x*+2, −*y*+2, −*z*+2; (iv) *x*, −*y*+3/2, *z*+1/2; (v) *x*, *y*, *z*−1; (vi) −*x*+2, −*y*+2, −*z*+1.
